# The Microtubule-Depolymerizing Kinesin-13 Klp10A Is Enriched in the Transition Zone of the Ciliary Structures of *Drosophila melanogaster*

**DOI:** 10.3389/fcell.2019.00173

**Published:** 2019-08-21

**Authors:** Veronica Persico, Giuliano Callaini, Maria Giovanna Riparbelli

**Affiliations:** ^1^Department of Life Sciences, University of Siena, Siena, Italy; ^2^Department of Medical Biotechnologies, University of Siena, Siena, Italy

**Keywords:** male gametogenesis, Klp10A, ciliary structures, transition zone, centrioles, *Drosophila*

## Abstract

The precursor of the flagellar axoneme is already present in the primary spermatocytes of *Drosophila melanogaster*. During spermatogenesis each primary spermatocyte shows a centriole pair that moves to the cell membrane and organizes an axoneme-based structure, the cilium-like region (CLR). The CLRs persist through the meiotic divisions and are inherited by young spermatids. During spermatid differentiation the ciliary caps elongate giving rise to the sperm axoneme. Mutations in Klp10A, a kinesin-13 of *Drosophila*, results in defects of centriole/CLR organization in spermatocytes and of ciliary cap assembly in elongating spermatids. Reduced Klp10A expression also results in strong structural defects of sensory type I neurons. We show, here, that this protein displays a peculiar localization during male gametogenesis. The Klp10A signal is first detected at the distal ends of the centrioles when they dock to the plasma membrane of young primary spermatocytes. At the onset of the first meiotic prometaphase, when the CLRs reach their full size, Klp10A is enriched in a distinct narrow area at the distal end of the centrioles and persists in elongating spermatids at the base of the ciliary cap. We conclude that Klp10A could be a core component of the ciliary transition zone in *Drosophila.*

## Introduction

Gametes are specialized cells that give rise to the offspring. Male and female gametes are produced by spermatogenesis and oogenesis occurring in testis and ovary, respectively. During spermatogenesis, the spermatogonial cells undergo mitotic divisions from which originate the spermatocytes that give rise to the haploid spermatids. Spermatids are round cells that differentiate during the final phase of spermatogenesis, called spermiogenesis, undergoing morphological changes to become mature sperm.

Several sperm dysfunctions cause infertility due to axoneme anomalies. Axonemal defects in primary cilia are often observed in human diseases, ciliopathies, in which genetic mutations affect ciliary assembly (Badano et al., 2006; Bisgrove and Yost, 2006; Baker and Beales, 2009; Nigg and Raff, 2009; Bettencourt-Dias et al., 2011; Waters and Beales, 2011; Inaba and Mizuno, 2015). In light of these observations, it appears of particular interest to study the mechanisms and the proteins involved in axoneme assembly.

The process of spermatogenesis shows fundamental similarities across the various phyla. For example, several cellular and regulatory mechanisms of spermatogenesis found in *Drosophila melanogaster* are conserved in other organisms, including humans. *Drosophila melanogaster* is a powerful system for studying the spermatogenesis and spermiogenesis processes, since it is relatively easy to examine the different stages involved in sperm development. The *Drosophila* testes consist of two close-ended tubules where the different stages of spermatogenesis are arranged in a chronological order. At the apical tip of the testis there is a cluster of somatic cells, the hub, that together with the Germline Stem Cells (GSCs) and the Somatic Stem Cells (SSCs) form the stem cell niche (Hardy et al., 1979). The hub is surrounded by 6–9 GSCs each associated with two SSCs. Each GSCs undergoes an asymmetrical division that originates two daughter cells: one cell remains in contact with the hub and maintains stemness, the other cell, called gonioblast moves away and begins differentiation (Yamashita et al., 2005). The gonioblast, enclosed by two non-dividing SCCs undergoes four mitotic divisions with incomplete cytokinesis leading to 16 spermatogones. These cells grow and become primary spermatocytes that produce 64 haploid spermatids at the end of meiosis (Fuller, 1993). The spermatids undergo spermiogenesis during which several cytological events transform the round spermatids into mature spermatozoa (Fabian and Brill, 2012).

Early spermatocytes inherit a centrosome that soon duplicates, so that at the beginning of the first meiotic prophase the germ cells hold two pairs of short centrioles composed of nine triplet microtubules and a central cartwheel (Riparbelli et al., 2009, 2012). The centrioles of each pair migrate toward the cell surface where each of them organizes the axoneme of a cilium like region (CLR) that protrudes from the cell membrane (Tates, 1971; Fritz-Niggli and Suda, 1972; Riparbelli et al., 2012). The centriole/CLR complexes increase in length during prophase progression; therefore, elongated centrioles and extended axonemes are found in mature spermatocytes (Gottardo et al., 2013). The CLRs remain throughout meiosis and are internalized with the centrioles to organize the spindle microtubules. The second meiotic division in *Drosophila* and most insects is not preceded by centriole duplication. Consequently, the secondary spermatocytes have only one centriole at each spindle pole. At the end of meiosis, the complex centriole/CLR is inherited by the spermatid and will be the precursor of the sperm flagellum (Gottardo et al., 2013). Since centrioles and CLRs are easily visible, they represent a very useful model for studying the localization of proteins involved in centriole and axoneme organization.

In addition to the CLRs found in male germ cells, *Drosophila* displays another type of ciliary structure, the sensory cilia that are associated with type-I sensory neurons (Gogendeau and Basto, 2010; Jana et al., 2016). Although, sensory cilia and CLRs are similar structures, their assembly relies on different mechanisms. Sensory cilia formation requires intraflagellar transport (IFT) mechanisms that are dispensable for CLR growth and sperm flagella elongation (Han et al., 2003; Sarpal et al., 2003; Avidor-Reiss and Leroux, 2015). The IFT-mediated process of axoneme assembly in canonical primary cilia depends on the transition zone (TZ), a specialized region at the junction between the centriole and the axoneme that is involved in cilia maintenance and compartmentalization (Reiter et al., 2012; Gonçalves and Pelletier, 2017) and is characterized by the *Y-*links, evolutionarily conserved structures that span the space between the doublet microtubules and the plasma membrane (Fisch and Dupuis-Williams, 2011; Czarnecki and Shah, 2012; Garcia-Gonzalo and Reiter, 2012).

The base of each sensory cilia in *Drosophila* displays *Y-*like structures (Vieillard et al., 2016; Jana et al., 2018) and contains some conserved TZ module proteins (Basiri et al., 2014; Pratt et al., 2016; Vieillard et al., 2016; Jana et al., 2018), suggesting that this specialized region can be regarded as a typical TZ. Remarkably, the CLRs of *Drosophila* spermatocytes contain the same TZ module proteins reported in sensory neurons (Basiri et al., 2014; Vieillard et al., 2016; Jana et al., 2018), but serial section analysis failed to reveal the presence of the typical *Y-*links (Gottardo et al., 2018). Therefore, the lack in *Drosophila* spermatocytes of a structured TZ, which in primary cilia represents a size-dependent diffusion barrier (Takao and Verhey, 2016; Garcia-Gonzalo and Reiter, 2017; Jensen and Leroux, 2017), points to the direct recruitment from the cytoplasm of the proteins required for CLR elongation, namely the cytosolic pathway of assembly (Avidor-Reiss and Leroux, 2015). However, despite the different assembly mechanisms, the growth of both CLRs and sensory neurons relies on the proper dynamics of the axonemal microtubules.

To gain insights in the organization of the microtubule scaffold during ciliogenesis we analyze the distribution of the kinesin-like protein Klp10A in type-1 sensory neurons and during *Drosophila* spermatogenesis, focusing our attention on the CLRs that form in male germ cells in the absence of IFT. *Drosophila* Klp10A is a microtubule-depolymerizing kinesin of the Kinesin 13 family. Kinesin 13 motors differs from the other kinesins in that they do not move along microtubules, but promote tubulin dimer disassembly, playing a key role in microtubule dynamics (Walczak et al., 1996; Desai et al., 1999). It has been proposed that the movement of the kinesin-13-specific loop-2 relative to the other areas of the kinesin-13–tubulin interface determines key conformational changes leading to tubulin bending and microtubule depolymerization (Benoit et al., 2018). Previous data reported that *Drosophila* Klp10A is involved in microtubule dynamics throughout interphase and cell division (Rogers et al., 2004; Goshima and Vale, 2005; Mennella et al., 2005) by affecting EB1 turnover (Do et al., 2014). Mutations in Klp10A lead to overly long centrioles in germ line and somatic *Drosophila* cells (Delgehyr et al., 2012; Franz et al., 2013; Chen et al., 2016). Moreover, the distribution of proteins involved in centriole assembly and function, such as *Drosophila* pericentrin-like protein (Dplp), Spd2, Sas4, and Sak/Plk4, is affected in the absence of Klp10A (Gottardo et al., 2016). We show here that in young spermatocytes the Klp10A protein is localized to the distal ends of centrioles that dock to the plasma membrane and is concentrated at the zone between the centriole and the CLR in mature spermatocytes. The Klp10A signal persists at the base of the ciliary cap in elongating spermatids. Moreover, this protein is enriched just above the distal centriole in sensory type-1 neurons. Our observations suggest that Klp10A could be a core component of the TZ of the ciliary structures in *Drosophila*.

## Materials and Methods

### Fly Stocks

The *Klp10A* mutant line was described in Peter et al. (2002) and the stock containing the Unc–GFP transgene in Baker et al. (2004). Oregon R flies were used as controls. Flies were raised on standard *Drosophila* medium at 24°C.

### Antibodies and Reagents

We used the following antibodies: mouse anti-acetylated tubulin (1:100; Sigma-Aldrich); rabbit anti-Spd2 (1:500; Rodrigues-Martins et al., 2007); mouse anti-Sas4 (1:200; Gopalakrishnan et al., 2011); and rabbit anti-Klp10A (1:300; Laycock et al., 2006). Staining of mutant *Drosophila* male germ cells with the antibody against Klp10A did not reveal appreciable signal (Delgehyr et al., 2012). The secondary antibodies used (1:800) Alexa Fluor-488- and Alexa Fluor-555-conjugated anti-mouse-IgG and anti-rabbit-IgG, were obtained from Invitrogen. Hoechst 33258, Paclitaxel (Taxol, from *Taxus brevifolia*), Dimethyl sulfoxide (DMSO), and Shields and Sang M3 Insect Medium were purchased from Sigma. Taxol was dissolved at 1 mg/ml in DMSO and stored frozen at −20°C.

### Immunofluorescence Preparations

Testes were dissected in phosphate buffered saline (PBS), squashed under a small cover glass and frozen in liquid nitrogen. After removal of the coverslip the samples were fixed in methanol for 10 min at −20°C. For antigen localization, the samples were washed 20 min in PBS and incubated for 1 h in PBS containing 0.1% bovine serum albumin (PBS-BSA, Sigma-Aldrich). The samples were then incubated overnight at 4°C with the specific antisera in a humid chamber. After washing in PBS-BSA the samples were incubated for 1 h at room temperature with the appropriate secondary antibodies. DNA was visualized after incubation of 3 min in Hoechst 33258 (1 μg/ml, Sigma-Aldrich). After rinsing in PBS the samples were mounted in 90% glycerol in PBS. Images were taken by an Axio Imager Z1 microscope (Carl Zeiss) equipped with an AxioCam HR cooled charge-coupled camera (Carl Zeiss). Gray*-*scale digital images were collected separately and then pseudocolored and merged using Adobe Photoshop 5.5 software (Adobe Systems).

### Drug Treatment

Testes were dissected in M3 medium from young pupae that contain cysts at the spermatogonial and spermatocyte stages. Testes were transferred in a 200 ml of M3 medium into a sterile 24-well plate. To assess the effect of microtubule stabilization on cilia length the dissected testes were incubated 24 h in M3 medium containing Taxol 5 mM. Controls testes were incubated in M3 medium containing DMSO alone. Specimens were fixed and stained following 24 h drug or DMSO treatments.

### Transmission Electron Microscopy

Testes and antennae from control and *Klp10A* pupae were dissected in PBS, and fixed in 2.5% glutaraldehyde in PBS overnight at 4°C. After washing in PBS, the samples were post-fixed in 1% osmium tetroxide in PBS for 1–2 h at 4°C. The material was then dehydrated through a graded series of ethanol, infiltrated with a mixture of Epon-Araldite resin and polymerized at 60°C for 48 h. Ultrathin sections were cut with a Reichert ultramicrotome, collected with formvar-coated copper grids, and stained with uranyl acetate and lead citrate. TEM preparations were observed with a Tecnai G2 Spirit EM (FEI) equipped with a Morada CCD camera (Olympus).

## Results

**FIGURE 1:**
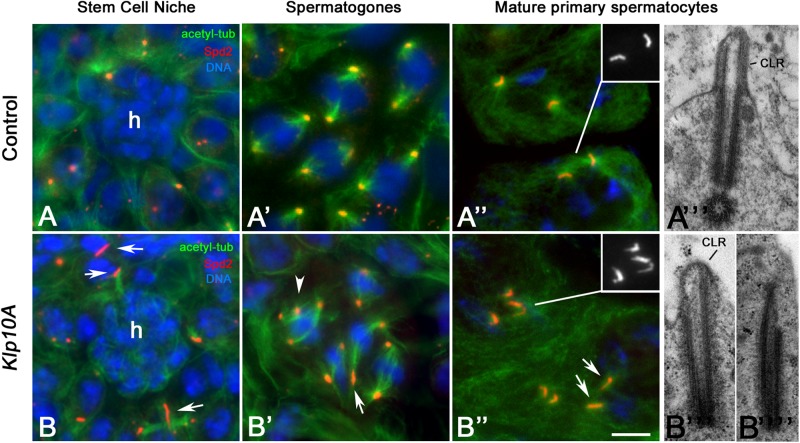
Centriole defects in *Klp10A* mutant testes. Centrioles are dot-like in germline stem cells **(A)** surrounding the hub (h) and spermatogones **(A’)** of control flies, becoming barrel-shaped in mature primary spermatocytes **(A”)** when organize a distinct cilium-like region (CLR) **(A”’)**. Mutant testes display irregularly sized centrioles (arrows) in stem cells **(B)** and spermatogones **(B’)** that often show additional centrioles with abnormal spindles (arrowhead, **B’**). Mature primary spermatocytes display elongated centrioles with short and abnormal CLRs **(B”’)** or centrioles that failed to dock to the plasma membrane and do not organize CLRs **(B””)**. Bar: 5 mm **(A–A”,B–B”)**; 0.3 mm **(A”’,B”’,B””)**.

Staining of control testes with an antibody against Spd2, a widely conserved centriole associated protein involved in centrosome organization (Varadarajan and Rusan, 2018), shows that dot-like centrioles were typically found in stem cells (Figure 1A) and spermatogones (Figure 1A’), whereas elongating rod-like centrioles were observed during spermatocyte maturation (Figure 1A”). Unusually long and short centrioles were observed in germ line stem cells (Figure 1B), spermatogones (Figure 1B’) and spermatocytes (Figure 1B”) of *Klp10A* testes. Abnormal centrioles were often associated with irregular mitotic (Figure 1B’) or meiotic (not shown) spindles. The centrioles of control early prophase spermatocytes moved to the cell surface to organize a CLR. Then, the CLRs elongated concurrently with the centrioles and reached their full dimensions in mature primary spermatocytes (Figure 1A”’). The abnormal shape of the *Klp10A* centrioles points to defects in CLR assembly. We find, indeed, that 64.4% of the centrioles (56/87) examined in mature primary spermatocytes at the ultrastructural level were elongated with reduced CLRs (Figure 1B”’). These CLRs were abnormal in shape reflecting the incomplete wall of the centrioles. 35.6% of the centrioles (31/87) found in late primary *Klp10A* spermatocytes undocked to the plasma membrane and were unable to organize distinct CLRs (Figure 1B””).

**FIGURE 2:**
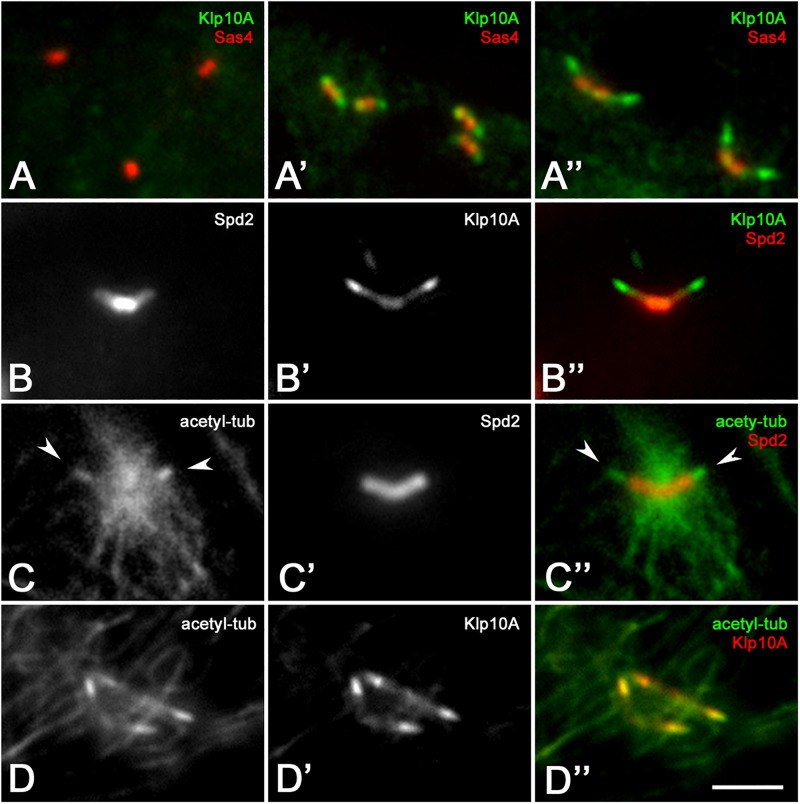
Klp10A is enriched at the distal end of the meiotic centrioles. Klp10A localization in spermatogones **(A)**, young primary spermatocytes **(A’)**, mature primary spermatocytes **(A”)**. **(B–B”)** Detail of a mature primary spermatocyte showing that Klp10A staining is weak along the centriole, but stronger at its distal tip. **(C–C”)** The axoneme of the CLRs (arrowheads) at the distal tip of the centrioles contained stable microtubules. **(D–D”)** The stronger Klp10A signal mostly colocalizes with the CLR. The number of stable microtubules increased at the base of the centrioles from prophase **(D)** to prometaphase **(C)** as the meiotic spindle starts to assemble. Bar: 3 mm.

Surprisingly, the Klp10A antigen was not detected on centrioles of the stem cell niche and spermatogones (Figure 2A) although abnormal centrioles were found at the beginning of spermatogenesis in mutant testes. Klp10A became apparent in young primary spermatocytes (Figure 2A’) but its distribution did not overlap the localization of the Sas4 antigen that was restricted to the basal region of the centriole. The gap between the localization of the two proteins increased in mature primary spermatocytes (Figure 2A”). The increased distance between the basal region of the centriole, as evidenced by the Sas4 labeling, and the distal localization of Klp10A might be correlated to the centriole elongation occurring during spermatocyte maturation. Double labeling with the centriole-associated Spd2 protein showed that the Klp10A antigen was mostly localized at the distal ends of the centrioles, the region where the CLR organized, although a faint distribution was also seen on the whole centriole (Delgehyr et al., 2012; Figures 2B–B”). Therefore, to uncover an eventual relationship between the spatial expression of Klp10A and the CLR, we performed a double labeling of control testes with the anti-Klp10A antibody and an antibody against acetylated-tubulin. This antibody mainly recognizes the stable microtubules associated with the axonemal structures of the flies, allowing us to easily detect the CLRs in primary spermatocytes (Figures 2C–C”). Immunofluorescence analysis revealed a strong Klp10A signal coincident with the CLRs (Figures 2D–D”).

### Klp10A Is Enriched on the Spermatocyte CLRs

**FIGURE 3:**
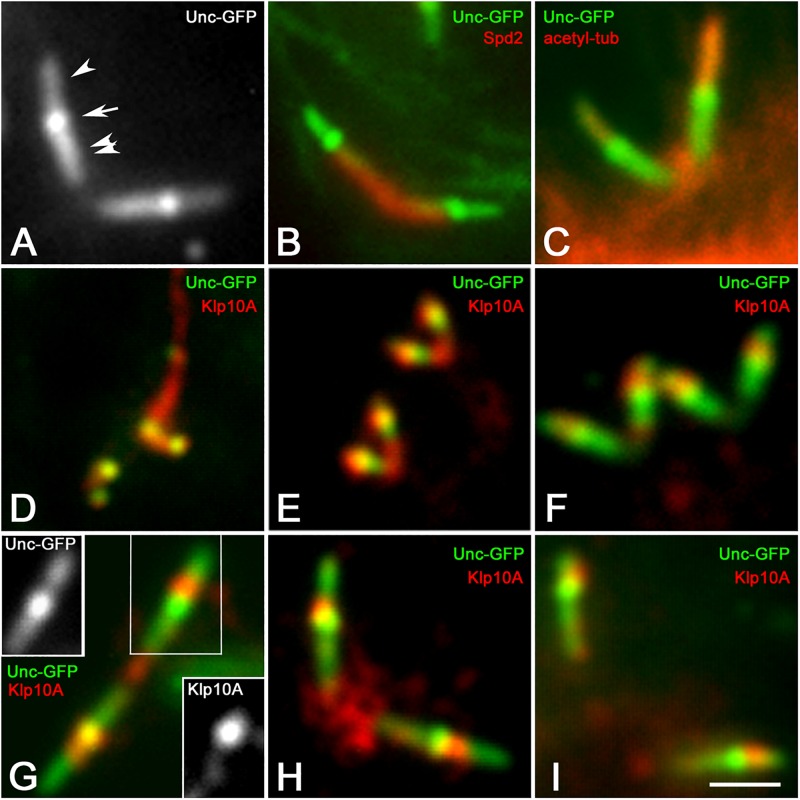
Klp10A signal is stronger in the transition zone of the CLRs. **(A)** Unc-GFP recognizes three distinct regions on the centriole/CLR of mature primary spermatocytes: a proximal region (double arrowhead), one intermediate region (arrow) and a distal region (arrowhead). Counterstain with the anti-Spd2 **(B)** and anti-acetylated tubulin **(C)** antibodies shows that Unc-GFP is localized along the centriole and the CLR, but neither of these antibodies recognize the Unc-GFP intermediate dot. **(D)** The Klp10A signal is first detected at the tip of the parent centrioles in young primary spermatocytes when a Klp10A filamentous structure is also observed at the base of one of the sister centriole pairs. The localization of Klp10A becomes restricted just above the intermediate Unc-GFP dot as the first prophase progresses **(E,F)** and is very distinct in mature primary spermatocytes **(G)**; insets in G are Unc-GFP and Klp10A separate channels showing a remnant Klp10A staining along the centriole. A cytoplasmic cluster of Klp10A is present between the parent centrioles during prophase **(G)** and increases in size during metaphase of the first meiotic division **(H)**; this cluster is very reduced during prophase of the second meiosis **(I)**. Bar: 2 mm.

To better delineate the localization of the Klp10A signal within the ciliary structures of the primary spermatocytes, we performed immunolocalization experiments on *Drosophila* spermatocytes expressing the Uncoordinated-GFP (Unc-GFP) fusion protein. The recruitment of Unc-GFP represents a useful tool to follow the assembly of the ciliary projections in the *Drosophila* spermatocytes. According to previous studies the Unc-GFP signal first appears in young spermatocytes as distinct dots when the centrioles reach the cell surface and start to organize the CLRs (Baker et al., 2004; Riparbelli et al., 2012). In mature primary spermatocytes the Unc-GFP signal became more complex and was found in three distinct regions of the centriole/CLR complexes (Figure 3A): one proximal region corresponding to the middle and the distal end of the centriole, one region overlapping the CLR, and a middle region between the centriole and the ciliary axoneme. Double labeling with the anti-Spd2 antibody shows that the Unc-GFP signal was not detected in the basal region of the centriole (Figure 3B), whereas it was very strong in the middle of the centriole/CLR complex (Figure 3A) a region that likely corresponds to the transition region between the centriole and the CLR (Vieillard et al., 2016). The distal Unc-GFP signal overlapped the ciliary axoneme recognized by the anti-acetylated-tubulin antibody (Figure 3C). A distinct Klp10A localization was first observed in young primary spermatocytes when the Unc-GFP signal appeared at the time of centriole-to-basal body conversion (Figure 3D). The Klp10A antibody also recognized filamentous structures that were closely associated with one of the sister centriole pairs (Figure 3D). It has been shown that these structures correspond to distinct microtubule bundles that extend into the peripheral cytoplasm of the polar spermatocytes from one of the mother centrioles of each pairs (Riparbelli et al., 2018). The spatial localization of Klp10A became distinct during prophase progression (Figures 3E,F) and was obvious in mature primary spermatocytes when the centriole/CLR complexes reached their full dimensions (Figure 3G). A feeble Klp10A signal was detected along the whole centriole, whereas a stronger labeling was observed just above the intermediate Unc-GFP dot (Figure 3G). This staining persisted during the further meiotic divisions (Figures 3H,I). A small cluster of Klp10A was observed between the centriole pairs during prophase progression (Figures 3D–G). This cluster increased in size at the poles of the first metaphase spindles (Figure 3H), but strikingly reduced during the second meiosis when the parent centrioles moved away (Figure 3I).

**FIGURE 4:**
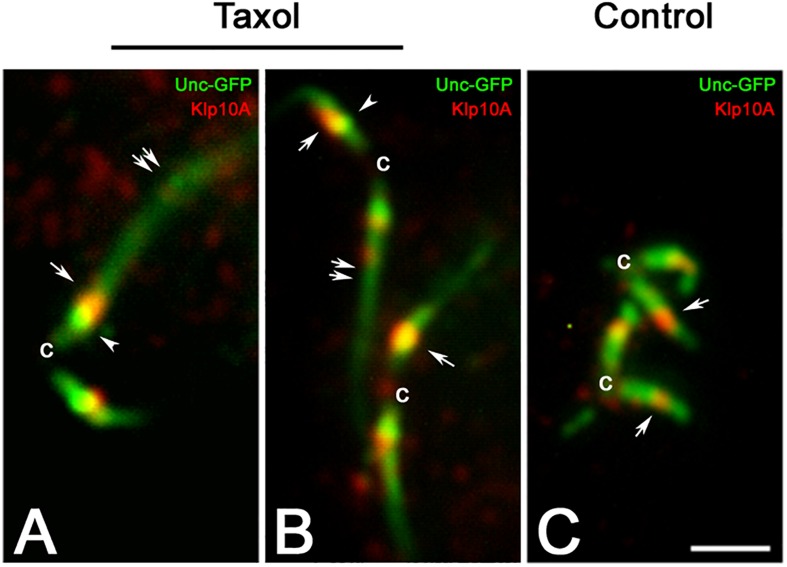
The localization of Klp10A does not change with the elongation of the CLR. Taxol treatment of young primary spermatocytes leads to the elongation of the CLRs **(A,B**, double arrows), but the Klp10A staining (arrows) maintains its localization close to the Unc-GFP intermediate dot (arrowheads) at the apical tip of the centrioles (c), as observed in control testes **(C)**. Bar: 2 mm.

It was previously showed that treatment of young primary spermatocytes with taxol leads to the dramatic elongation of the ciliary axoneme with the ensuing extension of the distal Unc-GFP domain (Riparbelli et al., 2013). However, the Unc-GFP localization at the centriole and at the TZ remains unmodified. The Klp10A distribution was also unchanged in taxol treated primary spermatocytes and the stronger signal was still localized close to the intermediate Unc-GFP dot, despite the CLR was unusually elongated (Figures 4A–C).

**FIGURE 5:**
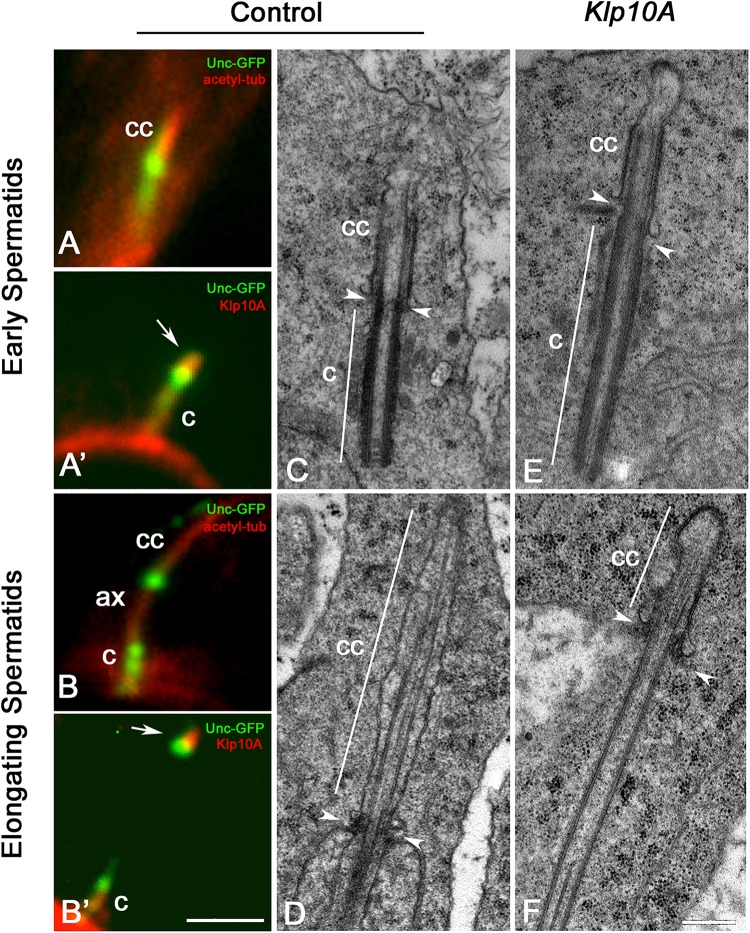
The ciliary cap is shorter in elongating Klp10A spermatids. The intermediate dot-like Unc-GFP signal is localized at the base of the ciliary cap (cc) in young spermatids **(A)** and moves away as the sperm axoneme (ax) elongates **(B)**. Klp10A (arrows) is associated with the intermediate Unc-GFP dot in early **(A’)** and elongating **(B’)** spermatids. Mutant young spermatids **(E)** have longer centrioles (c) compared to controls **(C)**, but elongating *Klp10A* spermatids **(F)** display ciliary caps (cc) shorter than controls **(D)**. Arrowheads mark the base of the ciliary cap in both control **(C,D)** and mutant spermatids **(E,F)**; note that in mutants the ciliary base is rather obliquely oriented. Bars: 2 mm **(A–B’)**; 0.4 mm **(C–F)**.

Each young spermatid inherits at the end of meiosis one centriole/CLR complex that represents the basis for the sperm axoneme formation. This structure look like the centriole/CLR complexes found during meiotic progression and also display a short axoneme (Figure 5A) and an intermediate Unc-GFP dot (Figure 5A). Klp10A was still observed next the Unc dot (Figure 5A’). At the onset of spermatid elongation, some Unc-GFP labeling was still associated with the centriole, whereas the Unc-GFP dot moved away from the distal end of the centriole concurrently with the elongation of the axoneme (Figure 5B; Gottardo et al., 2013). The Klp10A staining persisted close to the Unc-GFP dot (Figure 5B’).

EM analysis confirmed that the CLRs, or ciliary caps, inherited by the young control spermatids (Figure 5C) persisted at the apical end of the elongating sperm axoneme increasing two–three times their length (Figure 5D). Ciliary caps were also found in young (Figure 5E) and elongating (Figure 5F) mutant spermatids but their length was very reduced compared to controls. By contrast, the centriole was more prominent (Figure 5E). The basis of the invaginating cell membrane that surrounded the ciliary cap was associated with a small ring of dense material, the so-called “ring centriole” (Phillips, 1970) that in control spermatids was orthogonal to the axoneme (Figures 5C,D), whereas it was obliquely oriented in *Klp10A* mutant spermatids (Figures 5E,F).

### Reduced Klp10A Expression Results in Strong Structural Defects of Sensory Type I Neurons

**FIGURE 6:**
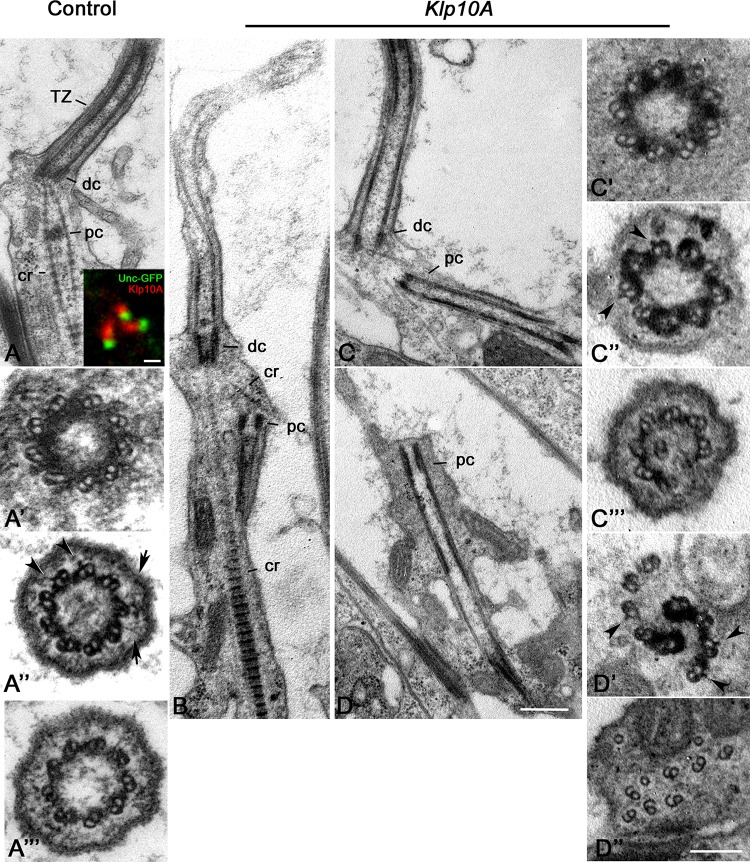
Ultrastructural defects in Johnston’s organs of *Klp10A* mutant flies. Control Johnston’s organs: Longitudinal section **(A)**; Cross section at the level of the distal centriole **(A’)**, the transition zone **(A”)**, the proximal region of the axoneme **(A”’)**. Klp10A is localized just above the Unc-GFP dot in sensory cilia (inset **A**). *Klp10A* Johnston’s organs: longitudinal sections of the whole ciliary complex **(B)**, proximal and distal centrioles **(C)** and proximal centriole alone **(D)**; cross section through the proximal centriole **(C’)**, the transition zone **(C”)** and the proximal region of the axoneme **(C”’)**; cross section throughout the intermediate **(D’)** and the distal **(D”)** regions of the proximal centriole. DC, distal centriole; PC, proximal centriole; CR, ciliary rootlet; TZ, transition zone; arrowheads, lateral projections emerging from the A-tubule; arrows, *Y-*links. Bars: 0.4 mm **(A–D)**; 1 mm (inset **A)**; 50 nm **(A’–A”’C’–C”’D’,D”)**.

For a better understanding of the Klp10A role in centriole/axoneme assembly, we examined the ultrastructure of the Johnston’s organ, a large array of sensory type I neurons in the antennae. Each unit, or scolopidium, of the Johnston’s organ usually displays two ciliary processes that assemble throughout a compartmentalized process of ciliogenesis (Avidor-Reiss and Leroux, 2015) and house at their base two short linearly arranged centrioles, one distal to the other. Unlike, primary spermatocytes, in which both the parent centrioles assemble a ciliary projection, only the distal centriole of the scolopidium templates the sensory ciliary axoneme (Figure 6A). The proximal centriole is shorter and enclosed within the rootlets emerging from the base of the distal centriole (Figure 6A). The distal centriole lacks a cartwheel and consists of nine doublet microtubules (Figure 6A’) that extend in a distinct TZ (Figure 6A”) and then in the ciliary axoneme (Figure 6A”’). Double labeling of transgenic flies expressing Unc-GFP, a protein specifically associated with the tips of the sensory dendrites where the distal centrioles are found (Baker et al., 2004; Enjolras et al., 2012), revealed that Klp10A was localized just above the Unc-GFP dot (Figure 6A, inset).

Seventy two percent (31; *n* = 43) of the ciliary structures of the mutant Johnston’s organs examined in longitudinal section were short with disorganized axonemal microtubules (Figure 6B). The rootlets that emerged from the base of the distal centriole were often disorganized and the proximal centriole was not positioned properly at the base of the cilia in several neurons (Figure 6B). Thus, proximal and distal centrioles lost their coaxial orientation. Distinct microtubules emerged from the proximal centriole and extended toward the basal cell cytoplasm to form elongated cylindrical structures often frayed at their extremities (Figures 6C,D). 66.6% of the distal centrioles examined in cross section (16; *n* = 24) look like control centrioles with nine microtubule doublets (Figure 6C’) whereas the remnant 34.4% (8; *n* = 24) had structural defects. However, 86% of the putative TZ examined (37; *n* = 43) showed discontinuities in the electron dense material associated with the axonemal microtubules (Figure 6C”). These discontinuities could also be observed in longitudinal sections (Figure 6C). Doublets of the ciliary axonemes were missing or misplaced (Figure 6C”’). Cross sections through the extension of the proximal centrioles often showed a disorganized wall in which we find naked doublets and doublets immersed in an electron dense material (Figure 6D’) reminiscent of the material observed through the TZ at the tip of the distal centrioles. Short lateral projections emerged from the A-tubules (Figure 6D’). Such lateral projections were also observed within the TZ of controls (Figure 6A”) and mutant (Figure 6C”) axonemes. Sections through the frayed extremities of the proximal centriole extensions revealed disorganized doublets with opened B-tubules (Figure 6D”).

## Discussion

The growth of the primary cilia in vertebrate cells is mediated by an IFT mechanism that requires specific carriers to move axonemal and ciliary membrane components from the ciliary base to its tip and back (Ishikawa and Marshall, 2017). Essential for the proper execution of this process is a specific region, the TZ that is restricted at the distal end of the basal body (Gonçalves and Pelletier, 2017).

*Drosophila* spermatocytes are characterized by surface protrusions, the CLRs, that look like primary cilia, but grow by IFT independent mechanisms (Han et al., 2003; Sarpal et al., 2003) and are not reabsorbed during cell division (Tates, 1971; Riparbelli et al., 2012). In contrast to basal bodies of conventional primary cilia that do not change length during ciliogenesis, the short centrioles of the young *Drosophila* spermatocytes elongate concurrently with the extension of the CLR axoneme (Fritz-Niggli and Suda, 1972; Gottardo et al., 2013). Because the centrioles of the *Drosophila* spermatocytes are continuous with the ciliary axoneme, they should not elongate. This aspect raises intriguing questions concerning the mechanisms of centriole elongation and the functional roles of the CLR.

The finding of conserved TZ proteins within the CLRs, namely Cep290, Chibby and components of the MKS complex (Enjolras et al., 2012: Basiri et al., 2014; Pratt et al., 2016; Vieillard et al., 2016) had led to the hypothesis that the whole CLR could be a modified TZ. However, TZ proteins are classically restricted to the basal region of the ciliary axoneme where *Y-*shaped links connect the axonemal doublets to the ciliary membrane. In contrast, *Drosophila* CLRs lack *Y-*links, consistent with the absence from *Drosophila* of the NPHP module proteins (Barker et al., 2014; Basiri et al., 2014) which are required to assemble the *Y-*links in primary cilia (Williams et al., 2011). We find *Y-*links like structures at the base of the sensory cilia confirming recent observations (Vieillard et al., 2016; Jana et al., 2018) that such links can also form in the absence of NPHP module proteins. However, typical *Y-*links usually arose from the space between the A and the B tubules (Szymanska and Johnson, 2012), whereas we find that in sensory *Drosophila* neurons the *Y-*links extend from the anterior margin of the A-tubule, raising questions on the actual similarity between these structures.

Unlike vertebrate cells in which the MKS module is crucial for cilia assembly and maintenance, sensory cilia lacking MKS proteins exhibit only subtle defects in the adult *Drosophila* flies (Pratt et al., 2016; Vieillard et al., 2016). Since the TZ of vertebrate primary cilia represents a selective gate that limits the transit of molecules within the ciliary compartment (Garcia-Gonzalo and Reiter, 2017; Jensen and Leroux, 2017), the absence of structured *Y-*links within the *Drosophila* CLRs points to a simple diffusion process of cytoplasmic proteins into the ciliary compartment, the so-called cytosolic pathway of assembly (Avidor-Reiss and Leroux, 2015). However, it is unclear how *Drosophila* TZ proteins are involved in modulating the axoneme assembly. The reduced expression of Cep290 led to abnormal and incomplete ciliary structures suggesting that this protein could be involved in the organization of the ciliary cap by enhancing the formation of a compartmentalized domain in which the axoneme tip would be organized (Basiri et al., 2014). Both the growth of the CLRs during spermatocyte maturation and the elongation of the sperm axoneme relies on microtubule assembly dynamics within the ciliary cap. It has been shown that mutations in the microtubule-depolymerizing Kinesin-13 *Klp10A*, lead to centriole and CLR defects during *Drosophila* male gametogenesis (Delgehyr et al., 2012; Gottardo et al., 2013). We show here that Klp10A is mainly localized to the basal region of the CLRs in the *Drosophila* spermatocytes just above the intermediate Unc-GFP dot. The distribution of Klp10A during male meiosis is strikingly similar to that of the TZ proteins Chibby and Cep290 (Enjolras et al., 2012; Basiri et al., 2014). During spermiogenesis Klp10A moves away from the apical tip of the centriole acquiring a localization like that of Cep290 and Chibby at the base of the ciliary cap. As in Cep290 mutants (Basiri et al., 2014), the centrioles of *Klp10A* primary spermatocytes are very elongated whereas the CLRs are reduced in length. These findings suggest that Klp10A may be a component of the TZ that ensures the proper organization of the axonemal doublets during CLR assembly. In the absence of Klp10A the behavior of the C-tubule is altered and the ciliary axoneme does not assemble properly, but very elongated centrioles are found (Gottardo et al., 2013). This suggest a balance between centriole and ciliary axoneme elongation, likely mediated by the direct action of Klp10A in controlling microtubule dynamics. The localization of Klp10a does not change with the extension of the ciliary axoneme after taxol treatment, suggesting that this protein performed its function in a stable and restricted defined region at the ciliary base. Klp59D, another kinesin-13 family member, is enriched within the whole CLR and along the ciliary cap of the elongating spermatids (Vieillard et al., 2016). However, unlike the short CLRs observed in *Klp10A* mutants, *Klp59D* spermatocytes display very elongated CLRs, suggesting that these kinesins perform opposite roles during the assembly of the spermatocyte TZ.

The early Klp10A signal has been found in young primary spermatocytes when the centrioles dock to the cell membrane and start to assemble the ciliary axoneme, although defects in centriole structure have been observed in GSCs and spermatogones. Chibby and Cep290 signals were also found at the time of centriole docking to the cell surface in primary spermatocytes (Enjolras et al., 2012: Basiri et al., 2014), but the structure of the centrioles during early stages of spermatogenesis was not investigated in these mutants. The discrepancy between the spatiotemporal appearance of the defects in centriole structure and the early detection of the Klp10A signal is still unclear. A cytoplasmic localization of Klp10A to the centrosomes has been observed in male GSCs (Chen et al., 2016), but a specific Klp10A signal on these centrioles has not been reported.

In control Johnston’s organs, two centrioles are typically aligned above the end of each dendrite. The distal centriole templates the ciliary axoneme that displays an elongated TZ. The proximal centriole is shorter and unable to nucleate a ciliary axoneme. Klp10A is localized at the distal ends of the sensory dendrites just above the Unc-GFP signal, a region that corresponds to the TZ (Enjolras et al., 2012). It has been shown that the TZ in auditory neurons consists of two regions and that the Unc-GFP signal is restricted to the proximal part of the TZ (Jana et al., 2018). Thus, our observations suggest that Klp10A could be specifically associated with the distal region of the TZ. Remarkably, the loss of *Klp10A* function leads to the extension of both the centrioles in opposite directions. This suggests that the coaxial centrioles in the Johnston’s organ could be disposed base to base. Alternatively, the unusual orientation of the centrioles could be due to the disruption of the rootletin cage thus enhancing the elongation of the proximal centriole toward the basal cytoplasm of the cell. It has been shown that reduced *Rootletin* function in chordotonal Johnston’s organs impairs ciliary rootlet assembly and the proximal centriole is displaced or missing, suggesting that rootlets may ensure the proper positioning of the proximal centriole within the base of the sensory cilium (Chen et al., 2015; Styczynska-Soczka and Jarman, 2015). Remarkably, the extensions of both the proximal and the distal centrioles display doublets surrounded by electron dense material as found in control TZ. We also observed short lateral projections associated with the A-tubules. Such projections are found together with the *Y-*links in the TZ of control Johnston’s organs. Therefore, the distal regions of the elongated centrioles in *Klp10A* Johnston’s organs may be equivalent to abnormally shaped TZs, even if they lack distinct *Y-*links. Our findings also suggest that the proximal centriole has the potential to assemble a TZ-like structure, a special skill until now ascribed to the distal centriole alone, unless the proximal centriole was converted in a distal one by the loss of *centrobin* (Gottardo et al., 2015). The observation that the proximal centriole starts ciliogenesis in Klp10A depleted Johnston’s organs is consistent with a critical role of this protein in controlling both proximal and distal centriole elongation. However, the proximal centrioles elongate to form TZ-like regions, but never ciliary structures are properly assembled. The phenotype we observed in mutant Klp10A Johnston’s organs is strikingly different from what has been found in mutant of other TZ proteins, such as Unc, Chibby and Cep290, in which both distal and proximal centrioles do not elongate (Baker et al., 2004; Enjolras et al., 2012; Basiri et al., 2014; Vieillard et al., 2016). Remarkably, all these TZ proteins are restricted or enriched to the proximal part of the TZ (Jana et al., 2018), whereas Klp10A is apparently enriched to the distal part alone.

We suggest that Klp10A can be regarded as a core component of the TZ in *Drosophila*, involved in the assembly and maintenance of the ciliary axoneme in male germ cells and chordotonal organs irrespective of the compartmentalized or cytosolic mechanisms of ciliogenesis.

## Data Availability

The raw data supporting the conclusions of this manuscript will be made available by the authors, without undue reservation, to any qualified researcher.

## Author Contributions

VP and MR performed all the experiments. GC and MR designed the experiments and wrote the manuscript.

## Conflict of Interest Statement

The authors declare that the research was conducted in the absence of any commercial or financial relationships that could be construed as a potential conflict of interest. The handling Editor declared a past co-authorship with one of the authors GC.
